# Weak interactions but potent effect: tunable mechanoluminescence by adjusting intermolecular C–H···π interactions†
†Electronic supplementary information (ESI) available. CCDC 1587616 and 1587617. For ESI and crystallographic data in CIF or other electronic format see DOI: 10.1039/c8sc01703d


**DOI:** 10.1039/c8sc01703d

**Published:** 2018-06-04

**Authors:** Zongliang Xie, Tao Yu, Junru Chen, Eethamukkala Ubba, Leyu Wang, Zhu Mao, Tongtong Su, Yi Zhang, Matthew P. Aldred, Zhenguo Chi

**Affiliations:** a PCFM Lab , GD HPPC Lab , Guangdong Engineering Technology Research Center for High-performance Organic and Polymer Photoelectric Functional Films , State Key Laboratory of Optoelectronic Material and Technologies , School of Chemistry , Sun Yat-sen University , Guangzhou , 510275 , P. R. China . Email: yutao33@mail.sysu.edu.cn ; Email: chizhg@mail.sysu.edu.cn ; Email: ceszy@mail.sysu.edu.cn

## Abstract

A new mechanoluminescent material (4-(diphenylamino)phenyl)(4-(diphenylphosphanyl)phenyl)methanone (CDpP), which displays tunable mechanoluminescent emission colors, has been designed and successfully synthesized. CDpP shows two distinct mechanoluminescent colors (blue and green) in different crystalline states. Single-crystal analyses and femtosecond transient emission studies reveal that the striking tunable mechanoluminescence properties of CDpP mainly originate from the different C–H···π interactions in the crystal structures. CDpP crystals with more C–H···π interactions show blue mechanoluminescence (ML), and the emission is attributed to the locally excited (LE)-state because the twisting process for the excited state is restricted by C–H···π interactions. Conversely, CDpP crystals with fewer C–H···π interactions display green ML, in which the red-shifted emission band originates from the twisted intramolecular charge transfer (TICT) excited state because the diphenylamine moiety is relatively free to rotate. The manipulation of weak intermolecular interactions in the crystalline state is a useful and reliable strategy for the tuning of the ML emission wavelengths.

## Abstract 2

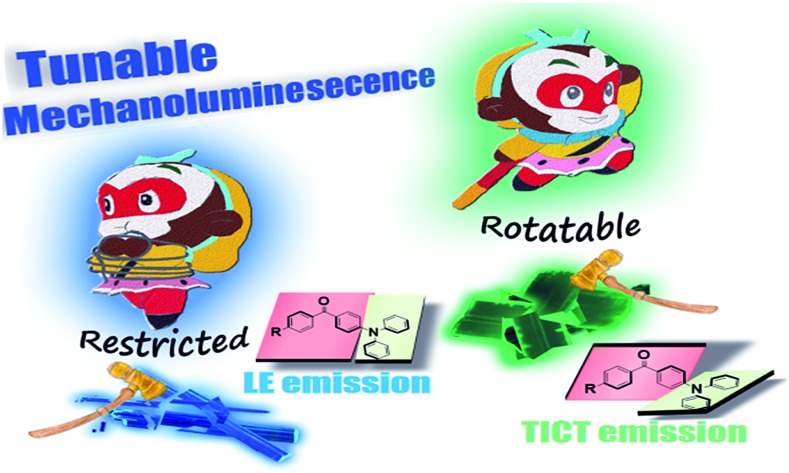
This study provides a reliable strategy to tune the mechanoluminescent colors by adjusting the weak intermolecular interactions.

## Introduction

Organic light-emitting materials have attracted much attention in both academic and industrial arenas for many years.^1^ The most common emission processes in organic materials are photoluminescence (PL, excited optically) and electroluminescence (EL, excited electrically). However, some types of organic materials exhibit ML, in which the emission occurs by mechanical actions, such as grinding, pressing or stretching, without the need for optical or electrical excitation.^2^ Diamond displays ML during fracture and polishing procedures^3^ and quartz shows ML during cutting with a diamond-impregnated saw blade^4^ and indeed when two quartz crystals are rubbed together. Some complexes based on different metal centers, such as Pt(ii), Mn(ii), Cu(i) and Au(i), also show intense ML properties.^5^ Although ML was first reported in 1605,^6^ only a handful of organic materials with such properties have been discovered.^7^ Examples of organic ML materials are *p*-anisidine,7*a* resorcinol,7*a* phosphine (phosphine oxide) derivatives,7b–d 9-anthracenemethanol,7*e* methyl salicylate,7*f**N*-phenyl imide,7g,h tetraphenylethene derivatives,7i,j borate derivatives,7k,l and others.7m–o Some non-luminescent materials, such as sucrose and tartaric acid, also generate an ML spectrum due to the emission caused by dinitrogen electrical discharge.^8^ Our group has previously reported ML from aldehyde- and sulfone-based materials, which exhibit blue-green and green ML under daylight at room temperature.^9^ Additionally, ML has also been observed in some aggregation-induced emission (AIE)-based,7i,^9^ thermally activated delayed fluorescence (TADF)-based,9*a* and phosphorescence-based materials.7k,m With great potential applications in real-time sensing for mechanical stress and material damage^10^ and light generators, which are driven by naturally vibrating mechanical actions.^11^ The development of mechanoluminescent materials with various emission colors is crucial.^12^ Jeong and co-workers successfully tuned the ML emission by changing the dopant ratios of two inorganic ML materials in the PDMS matrix.12*a* Strikingly, the ML color of the PDMS thin-films could also be manipulated by controlling the stress.12*a* Organic dyes were also doped in elastomeric zinc sulfide composites to achieve various ML colors.12*b* Besides, the ML colors and brightness could be tuned by changing the concentration of Mn^2+^ in the CaZnOS:Mn^2+^ salt.12*c* In contrast to traditional photoluminescent materials, the band-gap engineering of mechanoluminescent materials is more challenging because the ML properties are likely to perish after slight modifications of the molecular structure.7l–n Therefore, some strategies used to control the emission properties of traditional chromophores, such as controlling the π–π conjugation^13^ and tuning the electron donating/accepting abilities,^14^ cannot be applied to mechanoluminescent materials.

Internal molecular twisting processes have a significant impact on the intramolecular charge transfer (ICT) emissions.^15^ With different magnitudes of the twisting motions of the excited states, ICT emissions can be divided into two categories: LE-state emission (excited states are not obviously twisted during their formation) and TICT emissions (excited states are twisted during their formation).^15^,^16^ Tunable emission bands can be easily achieved by restricting or promoting the twisting processes by controlling the temperature, steric restrictions, and solvent polarity.^17^ Lately, the processes involved in the solid-state TICT emission have been reported and well studied by Chujo and other groups and tunable emission was demonstrated by controlling the twisting processes in the solid states.^18^ The TICT-based emission properties are related to intermolecular solid-state effects and, therefore, we presumed that the emission color might be simply switched by the manipulation of weak intermolecular interactions. If this strategy can also be realized in mechanoluminescent materials then the emission properties can be controlled by tuning the intermolecular interactions, rather than chemical structure modification, allowing ease of control.

**Fig. 1:**
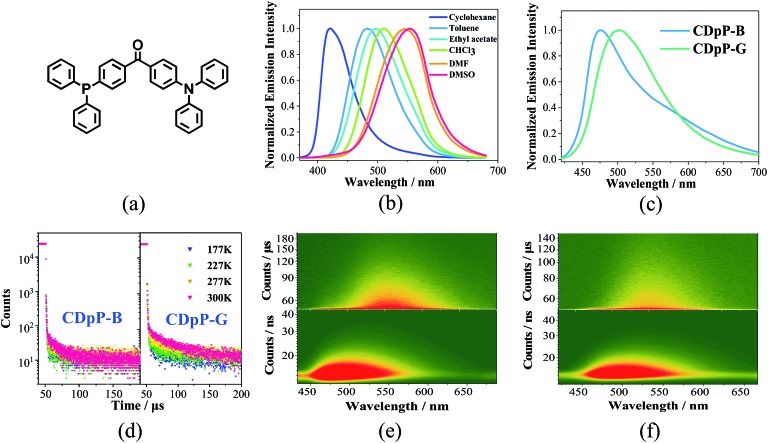
(a) Chemical structure of CDpP, (b) emission spectra of CDpP in various solvents with a concentration of 1 × 10^–5^ mol L^–1^, (c) emission spectra of CDpP in different crystalline states, (d) temperature-dependent emission decay spectra of CDpP-B at 473 nm (left) and CDpP-G at 504 nm (right), (e) time-resolved emission spectra of CDpP-B in nanosecond (below) and microsecond (above) ranges, and (f) time-resolved emission spectra of CDpP-G in nanosecond (below) and microsecond (above) ranges.

Many phosphine-containing compounds exhibit mechanoluminescence properties according to previous literature.7b–d and diphenylamine derivatives are typical TICT donor moieties.^19^ Therefore, a mechanoluminescent material with tunable emission bands might be achieved in an asymmetrical diphenylketone (acceptor) cored molecule flanked either side by triphenylphosphine (aiding ML properties) and diphenylamine (aiding TICT properties) moieties in a donor–acceptor type molecular system (CDpP). To satisfy this, herein we describe the synthesis of CDpP (Fig. 1(a)) and investigate its optical/photo-physical properties with crystal structure analysis. To our delight, two types of crystals were produced that showed different packing modes and distinct ML properties, in which the two types of crystals display sky-blue and green emission. To uncover the in-depth relationships between its unique ML properties and intermolecular interactions, various photophysical studies, single-crystal analyses and femtosecond emission studies were carried out. The relationships between the distinct ML colors of CDpP crystals (CDpP-B and CDpP-G) and different C–H···π interactions are revealed. We hope this study will provide a reliable strategy to tune the ML emission wavelengths by adjusting weak intermolecular interactions.

## Results and discussion

The UV-vis absorption spectra of CDpP in various solutions are shown in Fig. S4.† The lack of significant changes of the absorption maxima (*λ*_abs_) with changes in solvent polarity suggests a relatively non-polar ground state.^20^ Oppositely, Gaussian ICT emission bands can be detected for CDpP in different polarity solvents (Fig. 1(a)). Due to the solvent reorganization processes in the excited state, the emission maximum (*λ*_em_) of CDpP exhibits a large bathochromic shift from 421 nm to 553 nm with increasing solvent polarity (Fig. 1(b)).

Solvent-dependent spectral shifts of CDpP agree well with the Lippert–Mataga equation, which further demonstrates the ICT characteristics of the excited states (Fig. S5†). The details of the *λ*_abs_, *λ*_em_ and emission lifetimes (*τ*) in various solvents are listed in Table S1.† In addition, the *τ* of CDpP increases in polar solvents revealing the TICT emission properties according to previous reports.16b,^21^ More evidence for the twisting processes involved in the excited state of CDpP will be provided in the femtosecond emission studies *vide infra*.

Two different types of crystalline solids with different emission properties and powder X-ray diffraction (pXRD) patterns (Fig. S6†) were obtained using the vapor diffusion crystallization method with different recrystallization solvent systems. The crystals obtained from a dichloromethane–methanol mixed solvent system display sky-blue emission (named CDpP-B) with a *λ*_em_ of 473 nm, whilst the crystals obtained from a dichloromethane–hexane mixed solvent system show green emission with a *λ*_em_ of 504 nm (named CDpP-G). Different from the emission spectra in the solution state, both types of emissive crystals show non-Gaussian bands with prominent peak tailing (Fig. 1(c)). Due to the absence of the usual Gaussian distribution of an ICT emission peak other emission details at longer wavelengths were thought to be possible. In order to gain more insight into this, time-resolved emission studies of CDpP-B and CDpP-G were carried out (Fig. 1(e) and (f)). Emission properties with different emission origins are clearly detected for both types of crystalline powders. The higher-energy emission band observed in both samples are strong and dominant, which is assigned to the ICT emission, in which the differences in the emission properties of CDpP-B and CDpP-G are mainly attributed to this higher-energy ICT emission. The bathochromic shift (*ca.* 1300 cm^–1^) in emission for CDpP-G might be caused by TICT in the solid state, so in order to further investigate this we carried out femtosecond transient emission spectroscopy (*vide infra*). Temperature-dependent emission decay studies for the ICT emission bands of CDpP-B and CDpP-G were carried out and the results are shown in Fig. 1(d). The ICT emission of CDpP-G, compared to CDpP-B, shows more obvious delayed fluorescence components and stronger prompt processes by decreasing the temperature. Therefore, the ICT emission band for CDpP-G is consistent with typical TADF characteristics.^22^ With regard to CDpP-G, larger conformational distortion and complete charge separation during the formation of the charge transfer excited state are beneficial for narrowing the energy gap between S1 and T1 and, therefore, promoting TADF.^22^ These results are consistent with the aforementioned prediction that the ICT emission process for CDpP-G is from TICT, whilst the ICT emission band for CDpP-B originates from the LE-state (no obvious conformational distortion for the ICT excited state). The lower-energy emission bands of these two samples are quite similar in terms of both energy and lifetime. According to the previous literature, the lower-energy emission bands could be assigned to intermolecular charge transfer emission between neighbouring molecules.20*b* To further confirm the identity of the lower-energy emission band, TD-DFT calculations were performed for CDpP-B and CDpP-G both in the single molecular state and in the dimer state at the B3LYP/6-31G* level based on their single-crystal structures. All these results are listed in Tables S2 and S3 (ESI†). For the dimer states of both CDpP-B and CDpP-G, the HOMO and LUMO are located on separated neighbouring molecules, which indicates the possibility of intermolecular charge transfer. In the dimer state, the oscillator strengths for transitions between S0 and S1 are less than 0.01 for both CDpP-B and CDpP-G, and are much lower than that for transitions between S0 and S2. These results are in good accordance with the experimental data because the intermolecular charge transfer emission bands are much weaker than the intramolecular charge transfer bands in both the PL and the ML emission spectra.

**Fig. 2:**
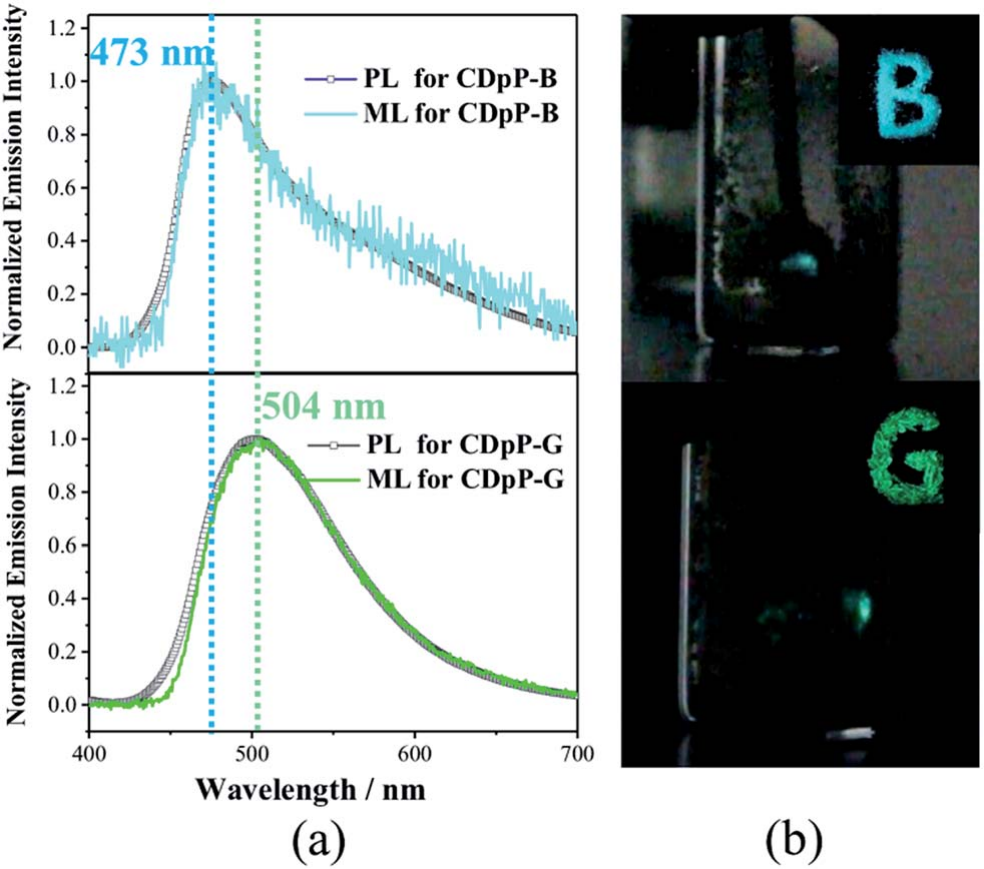
(a) PL spectra/ML spectra of CDpP-B (above) and CDpP-G (below), (b) photographs showing: (i) solid-state PL (top right) with letters “B” and “G” during irradiation with UV-light (365 nm), and (ii) generation of ML (the light spot) for CDpP-B (above) and CDpP-G (below) after pressing the powder with a spatula against the side-wall of a glass vial.

**Fig. 3:**
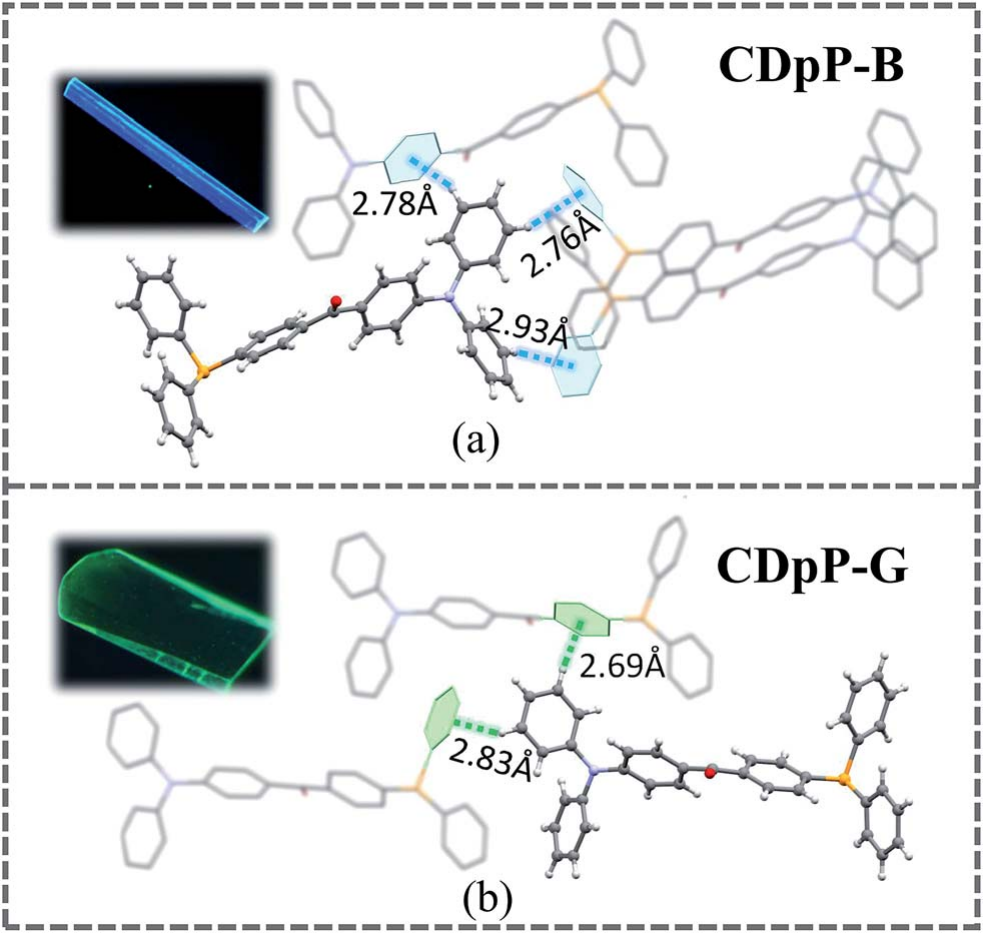
Single crystal structures of CDpP-B and CDpP-G with C–H···π intermolecular interactions involving the diphenyl moieties and luminescence pictures of the CDpP-B and CDpP-G single crystals in the dark under 365 nm UV-light irradiation.

ML studies were also performed on the crystalline powders of CDpP-B and CDpP-G. Both CDpP-B and CDpP-G exhibit strong ML with diverse properties for the two kinds of crystalline powders (Fig. 2). During grinding, CDpP-B displays strong blue ML emission (473 nm) and CDpP-G shows strong green ML emission (504 nm). Notably, the differences in ML properties are achieved for the same compound with different packing modes. To the best of our knowledge, this is the first report demonstrating different ML emission wavelengths of one compound with different “aggregation” states. In addition, the ML spectral characteristics of CDpP-B and CDpP-G are in accordance with their PL spectra. This indicates that these non-Gaussian ML spectral profiles, for both CDpP-B and CDpP-G, also consist of two different emission origins. Therefore, compound CDpP exhibits different ML properties by changing the packing modes and the ML displays dual-emission properties. The solid-state luminescent quantum yield of CDpP-B (31%) is a little higher than that of CDpP-G (26%). The higher luminescence quantum yield of CDpP-B might be attributed to more C–H···π interactions (see the single-crystal analyses *vide infra*), which could restrict the intramolecular rotations and vibrations of the excited states.^23^ On the contrary, the ML intensity of CDpP-B is obviously lower than GDpP-G (Fig. 3). With smaller average particle size and strengthened by more C–H···π interactions (see the single-crystal analyses *vide infra*), CDpP-B crystalline powders are more difficult to destroy than CDpP-G upon grinding or pressing actions. Therefore, the excited molecules of CDpP-B are fewer than that of CDpP-G under the same mechanical force. To demonstrate that the ML properties of CDpP are promoted by the diphenylphosphine moiety, a reported compound which lacks the diphenylphosphine unit from CDpP was synthesized according to previous literature.^24^ The emission properties of the reference compound in the amorphous state and in the crystalline state are shown in Fig. S7.† In the ML studies, the reference compound is non-emissive during the pressing and grinding processes. The comparison of ML spectra between CDpP-B, CDpP-G and the reference compound (Fig. S8†) further demonstrates that the reference compound does not exhibit ML properties. Therefore, the diphenylphosphine moiety in CDpP was quite necessary to achieve the ML properties.

Single crystals of both CDpP-B and CDpP-G were obtained by the vapor diffusion method using dichloromethane–methanol for CDpP-B and dichloromethane–hexane for CDpP-G. The CCDC numbers of CDpP-B and CDpP-G are 1587616 and ; 1587617, respectively. The single crystal analyses reveal that CDpP-B and CDpP-G molecules are constructed based on *P*2_1_/*n* and *Cc* space groups, respectively. In both cases several C–H···π interactions can be detected, in which the hydrogen atoms in the diphenylamine moiety are involved in the C–H···π interactions with the neighbouring molecules. The distances of the C–H···π interactions are 2.78 Å, 2.76 Å and 2.93 Å. Both the phenyl rings in the diphenylamine moiety are restricted by these C–H···π interactions. However, only two C–H···π interactions are formed between the hydrogen atoms of the diphenylamine moieties and phenyl rings in neighbouring molecules for each molecule in the CDpP-G crystal structure. The distances of the C–H···π interactions are 2.69 Å and 2.83 Å. In CDpP-G crystals, only one phenyl ring in the diphenylamine moiety is restricted. More C–H···π interactions result in more restricted intramolecular rotations of the diphenylamine units, and therefore, internal rotations in the CDpP-B crystal are more restricted compared to the rotations in the CDpP-G crystals. Therefore, the CDpP-B crystal is prone to display LE-state emission with less distortion of its excited state. Further demonstration is shown by femtosecond emission studies *vide infra*. With different C–H···π interactions involving the diphenylamine units, the ML spectral wavelengths are quite diverse for the same molecular structure. The planarization between two phenylene groups across the carbonyl group is also an important factor to affect emission and ML properties. The dihedral angles between the two phenylene groups across the carbonyl group for CDpP-B and CDpP-G are shown in Fig. S9 in the ESI.† The difference between the dihedral angles in the two crystals is quite small (55.3° for crystal CDpP(B) and 55.0° crystal for CDpP(G)). Thus, it is further confirmed that the different emission properties between the two types of crystals originated from the different C–H···π interactions other than the different dihedral angles.

**Fig. 4:**
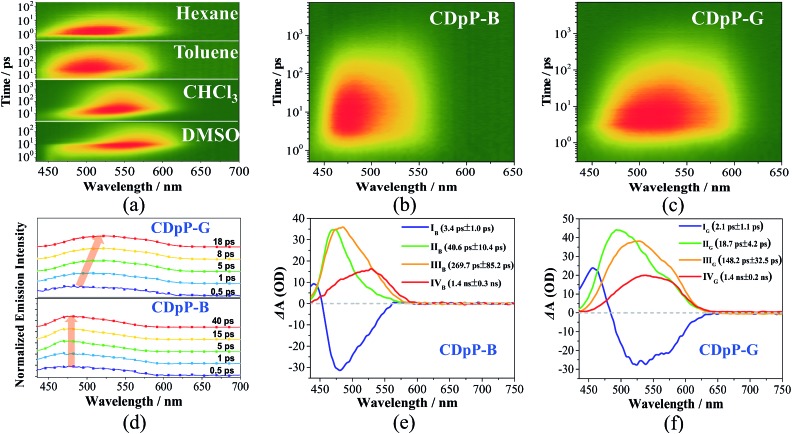
(a) Femtosecond transient emission spectra of CDpP in various solvents at a concentration of 1 × 10^–5^ mol L^–1^, (b) femtosecond transient emission spectra of CDpP-B crystals, (c) femtosecond transient emission spectra of CDpP-G crystals, (d) normalized time-resolved emission spectra of CDpP-B (below) and CDpP-G (above) upon excitation at 400 nm and gating at the indicated delay times, (e) spectral distributions of the pre-exponential coefficients obtained from a global analysis of the femtosecond transient emission spectrum of CDpP-B, and (f) spectral distributions of the pre-exponential coefficients obtained from a global analysis of the femtosecond transient emission spectrum of CDpP-G.

**Fig. 5:**
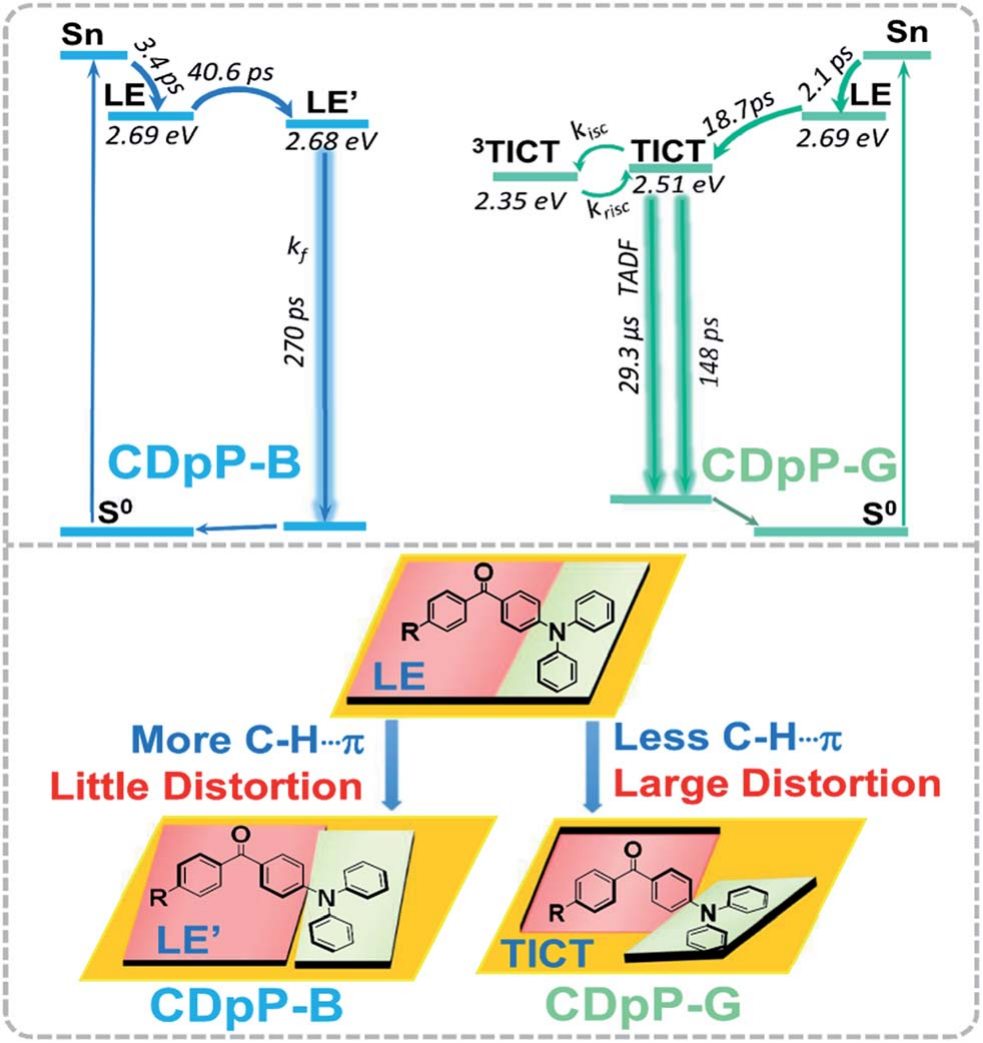
Photodynamic schemes of the emission processes of CDpP-B and CDpP-G (above) and the illustration of the different photodynamic processes for these two types of crystals (below).

As mentioned previously, the ML spectra of CDpP-B and CDpP-G are in accordance with their PL spectra with respect to spectral shape and emission maxima, respectively. Therefore, their ML emission and PL emission for each type of crystal have the same origin. Thus, the ML dynamic processes of CDpP-B and CDpP-G could be studied according to their PL processes. Excited-state dynamics in the femtosecond regime can be studied by femtosecond emission spectroscopy, which can reveal the formation and relaxation processes of the excited states. Here, a short laser pulse operates as a gate for the detection of emitted light and only the fluorescence light arriving at the detector at the matching time as the gate pulse is detected. Fig. 4(a) shows the femtosecond transient emission spectra of CDpP in various degassed solutions. The emission maxima in all solvents show red-shifts within 100 ps during their emission processes, which is assignable to the relaxation processes of the excited states.^25^ In polar solvents (CH_3_Cl and DMSO) the red-shifts are much more obvious compared to non-polar solvents (hexane and toluene) and reveal that CDpP is prone to show TICT emission with large conformation distortion in polar solvents.16b,^21^ Femtosecond transient emission spectra of the crystals of CDpP-B and CDpP-G are shown in Fig. 4(b) and (c). For CDpP-B, the emission maxima are almost maintained during the emission process, however, in contrast CDpP-G shows obvious red-shifted emission peaks during the emission process. To clearly reveal the difference in emission maxima changes between these two types of crystals, selected normalized transient emission spectra of CDpP-B and CDpP-G are shown in Fig. 4(d). Global fitting analyses were performed according to the transient emission spectra of CDpP-B and CDpP-G and the spectral distributions of the pre-exponential coefficients are shown in Fig. 4(e) and (f). Accordingly, the femtosecond transient emission spectra of both CDpP-B and CDpP-G were fitted satisfactorily with a model consisting of four species (species I_B_ to IV_B_ for CDpP-B and species I_G_ to IV_G_ for CDpP-G). The decay lifetimes for each species are also shown in the spectra. The fastest decay species (I_B_ and I_G_) with lifetimes equal to 3.4 and 2.1 ps for CDpP-B and CDpP-G represent the formation processes of an initial ICT-state (LE-state) according to previous reports.^26^ These spectra with positive values in the higher-energy range and negative values in the lower-energy range match well with the relaxation processes from higher-energy levels to the LE-states.^26^ Species IV_B_ and IV_G_ with decay lifetimes of more than 1 ns for these two samples could be ascribed to the intermolecular charge transfer emissions due to the similarity between the spectral distributions of the pre-exponential coefficients and the steady emission spectra for the intermolecular charge transfer emission bands. The decay processes II_B_ and II_G_ with lifetimes for CDpP-B and CDpP-G of 40.6 ps and 18.7 ps, respectively, are tentatively assigned to the relaxation processes of the LE-state. The decay lifetimes of these processes are also in agreement with the lifetimes of the emission maximum red-shift processes of these two samples (Fig. 4(d)). Species III_B_ and III_G_ for these two samples are in line with the emission processes related to the higher-energy ICT bands both in spectra and in decay lifetime. Therefore, the differences between species II and III reveal the magnitude of the conformational changes that occur during the relaxation processes of the LE-states. For CDpP-B, only small changes can be detected between the species II_B_ and III_B_ (Fig. 4(e)). The ICT emission mainly originates from LE-states with a slight relaxation process (named LE′). In contrast, an obvious red-shift between species II_G_ and III_G_ demonstrates the twisting motion during the relaxation processes of the LE-state to decrease its energy before the emission process (Fig. 4(f)). Therefore, it can be concluded that the diverse emission properties of CDpP-B and CDpP-G are mainly ascribed to the differences in the conformational changes during the relaxation processes of the LE-states. The blue emission band for CDpP-B originates from the LE-state with minor conformational changes during the relaxation processes, while the green emission for CDpP-G features a typical TICT emission process. In accordance with their relative crystal structures, the molecular twisting processes in the excited state of crystal CDpP-B are more difficult compared to crystal CDpP-G, due to the more C–H···π interactions involving the diphenylamine moieties. In addition, the decay lifetime of the relaxation process for CDpP-B (40.6 ps) is longer compared to CDpP-G (18.7 ps), which further demonstrates that the decay processes might be more difficult in CDpP-B due to more C–H···π interactions. To clearly reveal the different emission processes involved in CDpP-B and CDpP-G, the photodynamic schemes for these two types of crystals are shown in Fig. 5. The details of the energy level conformation for the photodynamic scheme are shown in the ESI (Fig. S12†).

## Conclusions

To conclude, a new mechanoluminescent material CDpP has been designed and synthesized. CDpP displays two distinct ML emission colors with emission maxima of 473 nm (CDpP-B) and 504 nm (CDpP-G) for two distinct crystalline states, which is unique compared to previously reported mechanoluminescent materials. Photophysical studies reveal that the non-Gaussian ML for CDpP-B and CDpP-G originates from the ICT excited states mixed with intermolecular charge transfer characteristics. Single-crystal analyses and femtosecond emission studies for these two types of crystals demonstrate that the ML properties of the crystals are distinct and mainly originate from the relaxation processes of their ICT excited states. Restricted by more C–H···π interactions, CDpP-B shows LE-state emissions with minor conformational changes during the relaxation processes. In contrast, CDpP-G is prone to display red-shifted TICT emissions due to the relatively free diphenylamine moieties with fewer C–H···π interactions. This study not only provides a rare example of achieving two kinds of ML emission colors based on one compound, but also suggests a reliable strategy to tune the ML emission wavelengths by manipulating weak intermolecular interactions.

## Conflicts of interest

There are no conflicts to declare.

## Supplementary Material

Supplementary information

Crystal structure data
