# Supplementary material for: Weak interactions but potent effect: tunable mechanoluminescence by adjusting intermolecular C–H···π interactions
Source: Chem Sci. 2018 Jun 4;9(26):5787–94. doi: 10.1039/c8sc01703d (PMC6050600; doi:10.1039/c8sc01703d)
Supplement: Supplementary file 1 [file SC-009-C8SC01703D-s001.pdf]

Electronic Supplementary Material (ESI) for Chemical Science.
This journal is © The Royal Society of Chemistry 2018

Supporting Information

1 Synthetic details

The synthetic procedure for CDpP is listed in scheme S1 and the experiment details were in the following section. Compound CFI and CDpI were synthesized according to the previous literature.[1] The chemical structures for these intermediate compounds were determined by ^{1}H NMR spectroscopy and high resolution EI mass spectroscopy. Final compounds were confirmed by ^{1}H NMR spectroscopy, $^{31}P\{^{1}H\}$ NMR spectroscopy, elemental analysis and high resolution EI mass spectrometry.

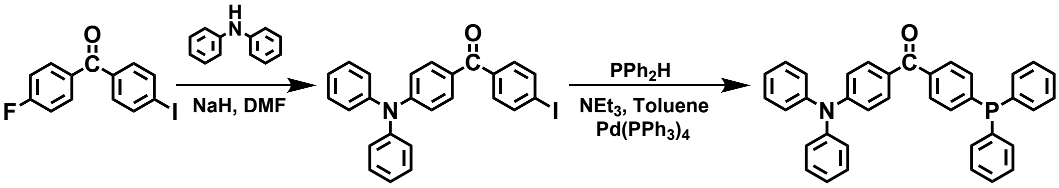

CFI CDpI CDpP

Scheme S1 Synthetic routes for compounds 2ThDp and 2ThDpF; (b) Synthetic routes for compounds 3ThDp and 3ThDpF.

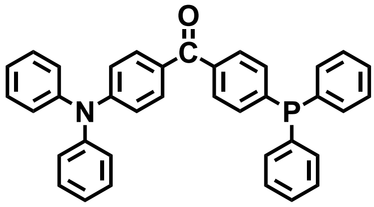

(4-(diphenylamino)phenyl)(4-(diphenylphosphanyl)phenyl)methanone (CDpP)

Diphenylphosphine in hexane solution (1.0 M, 1.1 mL) was added in a dropwise manner to a mixture of CDpI (475.3 mg, 1.0 mmol), $Pd(PPh_3)_4$ (23.1 mg, 0.02 mmol) in degassed toluene (30 mL) and Et_3N (9 mL) solution, under an argon atmosphere. After refluxed and stirred for 12 hours, the resulting mixture was filtered and the filtrate was collected. The filtrate was poured into water and extracted with CH_2Cl_2. Further purification was carried out by chromatography on silica gel with CH_2Cl_2-hexane (1:1, v/v) as eluent. Yield: 432.2 mg (81.0%). ^{1}H NMR (500 MHz, deuterated DMSO, 298 K): δ = 7.65-7.69 (m, 4H), 7.29-7.44 (m, 16H), 7.18-7.22 (m, 6H), 6.87-6.89 (d, 8.9 Hz, 1H). $^{31}P\{^{1}H\}$ NMR (deuterated DMSO, 298 K): δ = -5.13. High solution EI-MS:

m/z found: 533.1901 [M]$^+$; calcd for $C_{37}H_{28}NOP$: 533.1909. Elemental analyses (%) calcd for $C_{37}H_{28}NOP$: C 83.28, H 5.29, N 2.62; found: C 82.91, H 5.60, N 2.41.

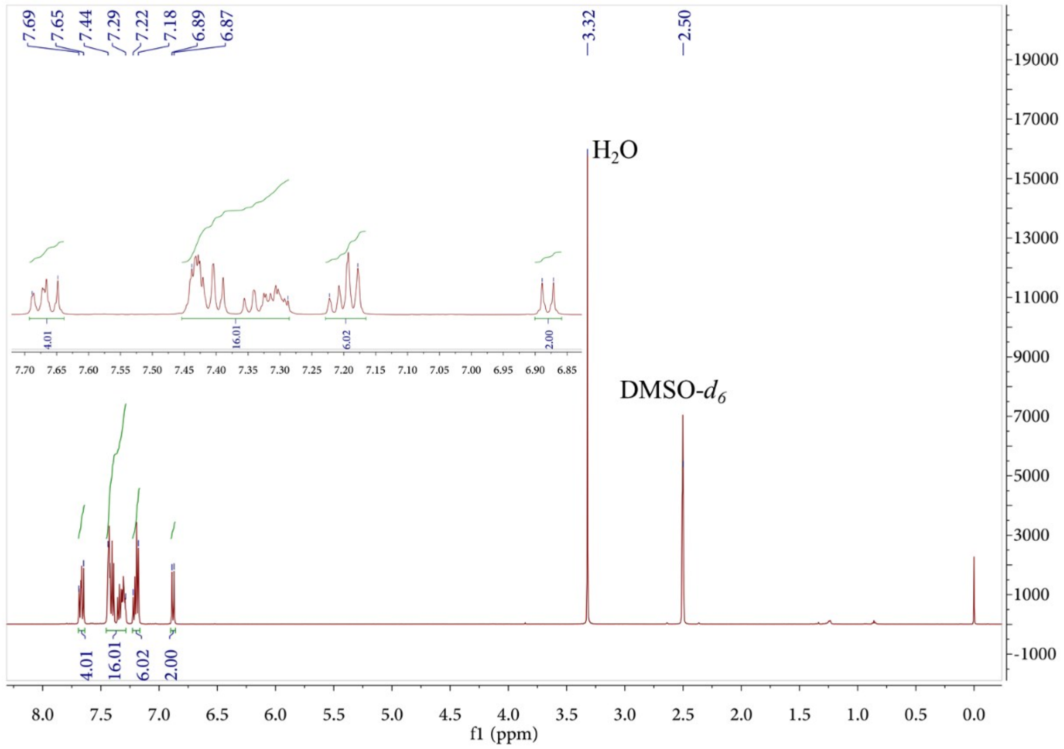

Figure S1 ^{1}H-NMR spectrum of compound CDpP.

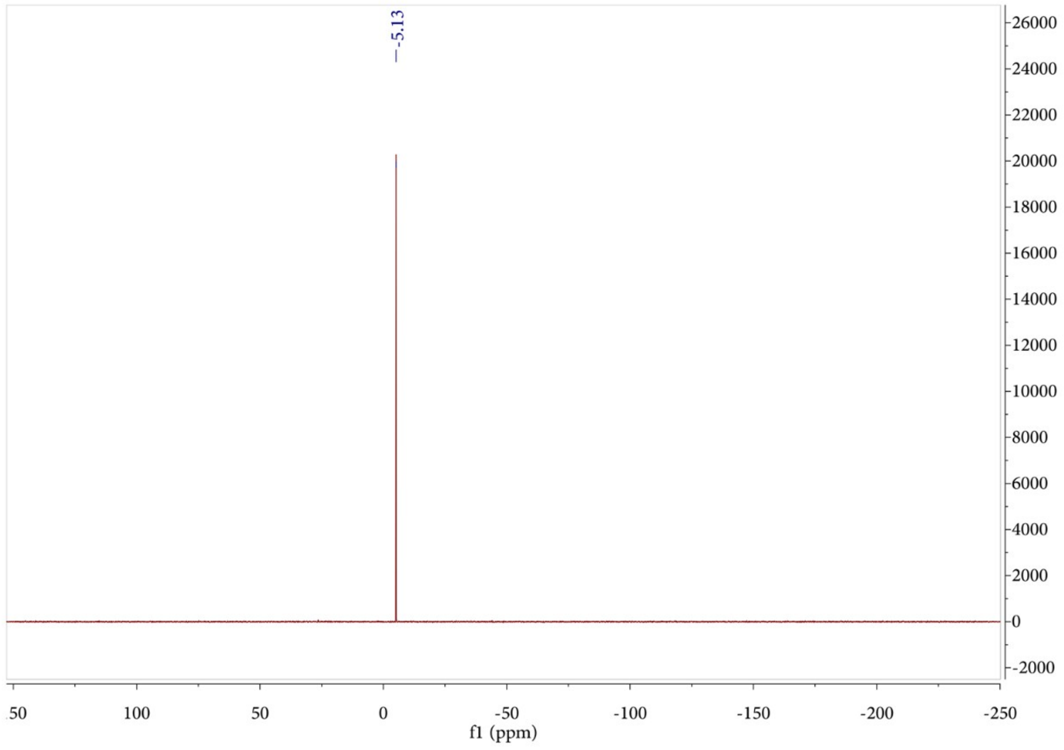

Figure S2 $^{31}P\{^1H\}$ NMR spectrum of compound CDpP.

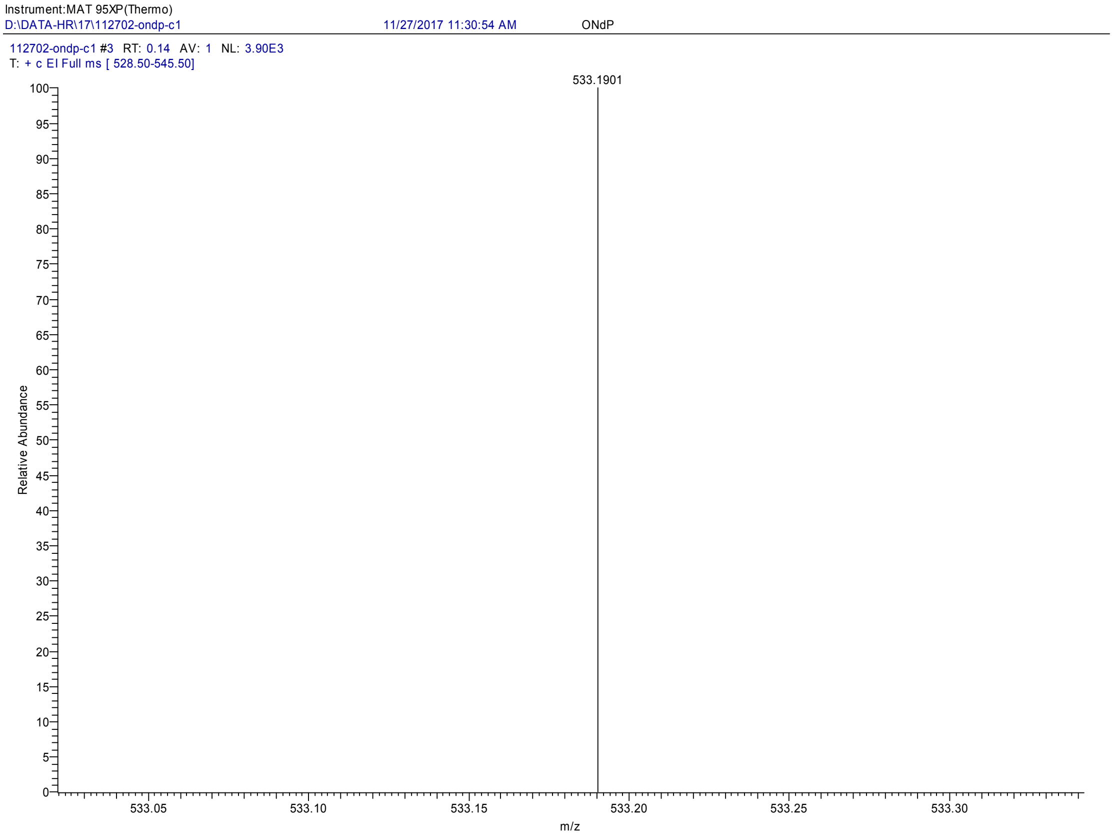

Figure S3 High Resolution EI mass spectrum of compound CDpP.

2 Physical measurements and instrumentations

^{1}H NMR and $^{31}P\{^{1}H\}$ NMR spectra were measured on a Bruker Avance III 500 NMR (Nuclear Magnetic Resonance Spectrometer). For ^{1}H NMR, the chemical shifts were relative to tetramethylsilane (TMS). Positive ion EI mass spectra were obtained on a Thermo MAT95XP high resolution mass spectrometer. The elemental analysis studies were performed with a Vario EL analyzer. Single-crystal X-ray analyses were conducted with an Oxford Diffraction Germini S Ultra X-ray Single Crystal Diffractometer using a (Cu) X-ray source. UV-vis absorption spectra were achieved on a UV-vis spectrometer (Hitachi U-3900). Steady state fluorescence and time-resolved emission studies (nano-second ranged) were carried out on a Horiba Scientific Fluorolog-3 spectrofluorometer. Femtosecond spectroscopies for samples were measured with the commercial fluorescence upconversion setup (Halcyone from Ultrafast Systems). The laser source consists of an ultrafast amplified Ti:Sapphire laser

with a pulse length of 95 fs, a central wavelength of 800 nm and a repetition rate of 1 kHz. One part of the output beam was used to pump an optical parametric amplifier (TOPAS from Light Conversion/Newport-Spectra-Physics) as the source for the pump pulse, a wavelength of 400 nm and a pulse length of 140 fs. After the quartz cuvette the pump pulses were filtered off by a 400 nm long-pass filter.

3 Photophysical data and spectra

Table S1. Absorption maxima, emission maxima and emission lifetimes in various solvents for CDpP.

Solvent	v_a (nm)	v_f (nm)	$\bar{v}_a$ - $\bar{v}_f$ (nm^{-1})	Δf	τ (ns)
C-hexane	363	421	3.79524E-4	-0.001	2.86
Toluene	371	474	5.85713E-4	0.013	2.87
EtOAC	365	495	7.19524E-4	0.171	3.60
TCM	378	510	6.84718E-4	0.149	3.58
DMF	370	544	8.64467E-4	0.275	5.67
DMSO	371	553	8.871E-4	0.265	6.27

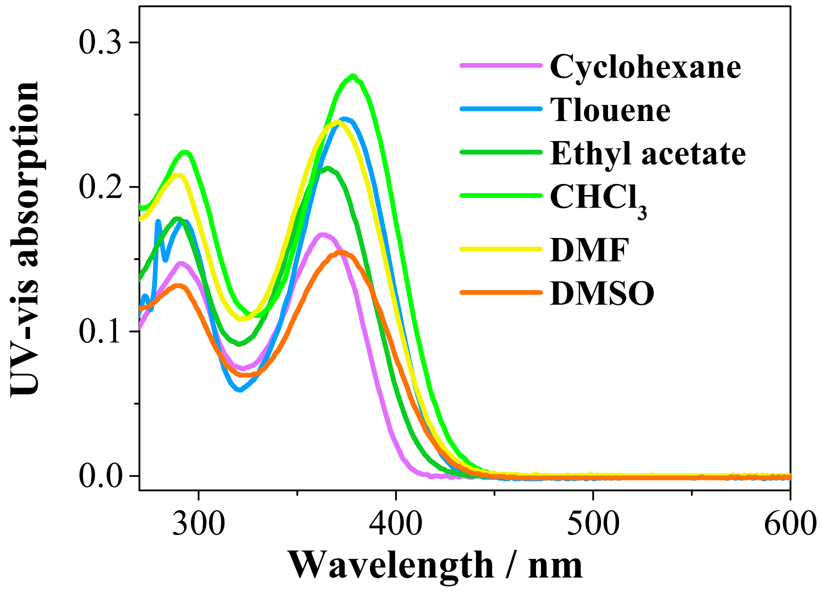

Figure S4 UV-vis absorption spectra for CDpP in various solutions.

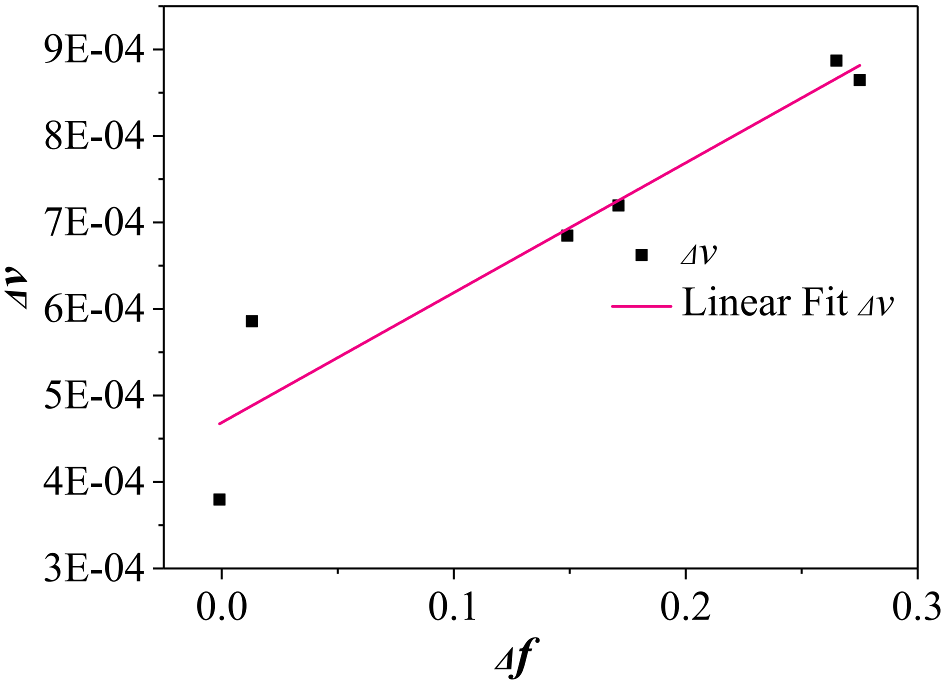

Figure S5 Lippert–Mataga plot of CDpP in solvents of increasing polarity.

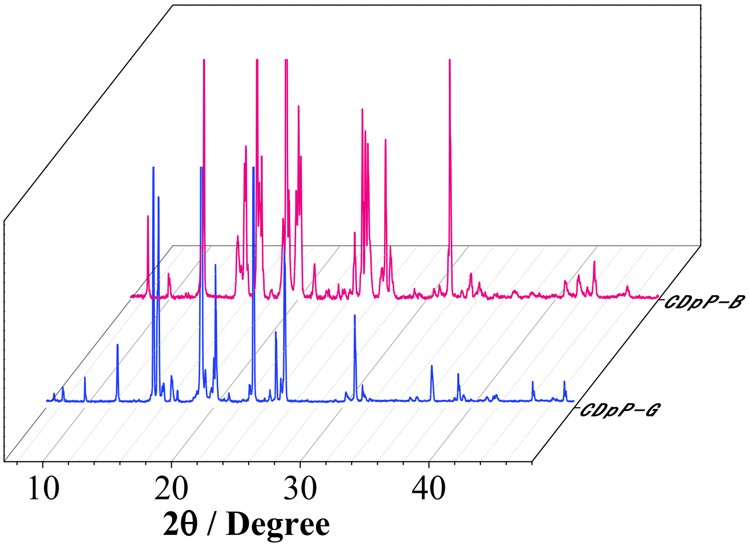

Figure S6 pXRD spectra for CDpP-B and CDpP-G

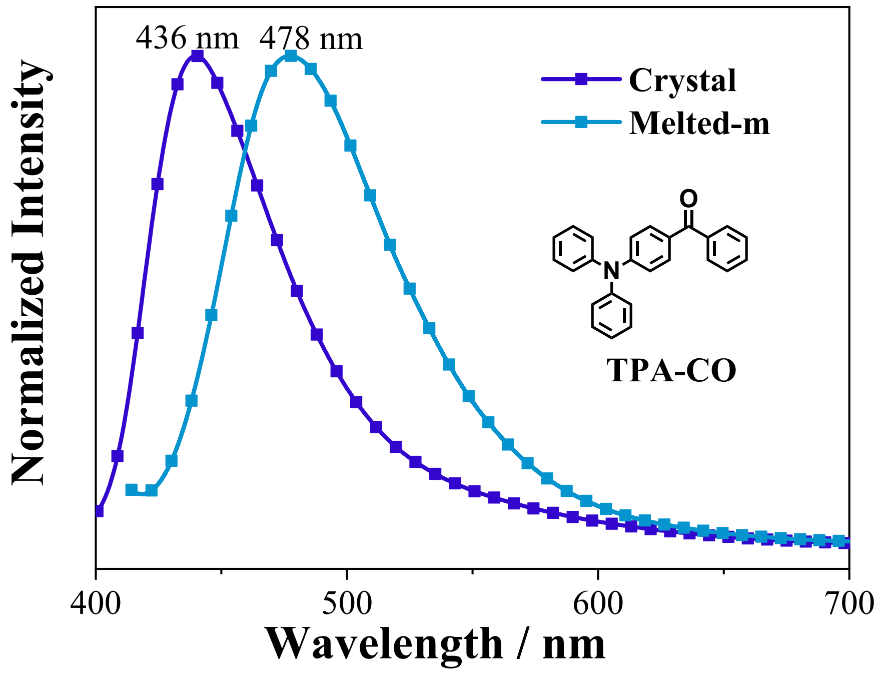

Figure S7 Emission spectra for TPA-CO in crystalline and Melted-m (amorphous) states.

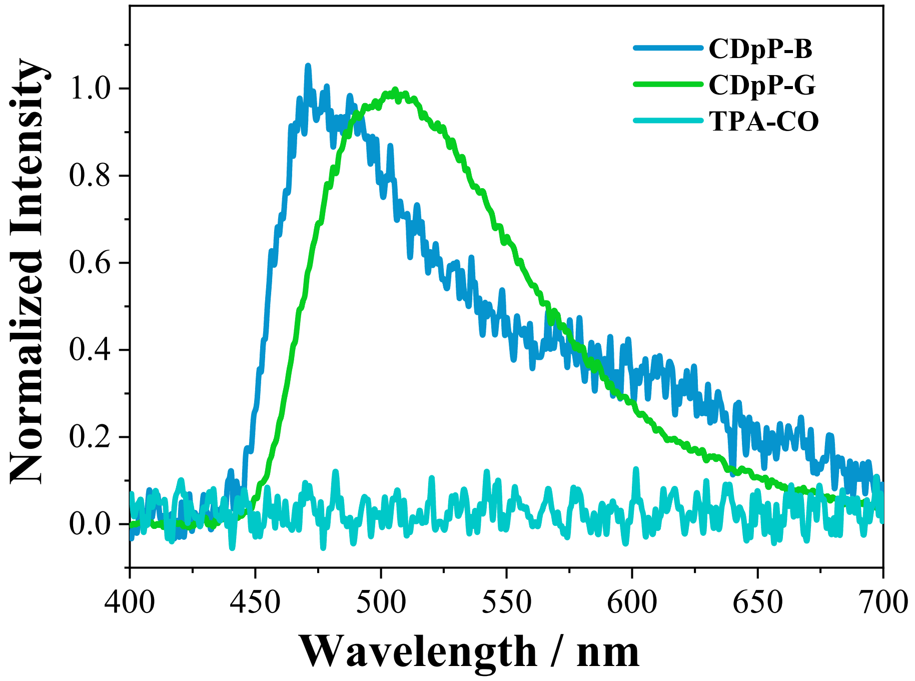

Figure S8 ML spectra for TPA-CO (in crystalline state), CDpP-B and CDpP-G.

4 Computational Details

Calculations were performed for monomer and dimer of CDpP-B and CDpP-G according to their single-crystal structures with B3LYP/6-31G* level. The distributions of HOMO, LUMO for their single molecular states and dimer states were listed in Table S2

Table S2. Calculated HOMO and LUMO distributions for CDpP-B and CDpP-G both for monomer states and their dimer according to their single crystal structures.

**Sample**	LUMO+1	LUMO	HOMO	HOMO-1
CDpP-B	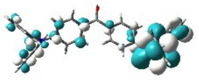	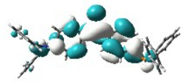	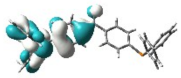	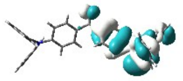
CDpP-B(dimer)	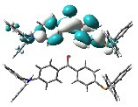	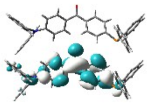	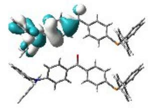	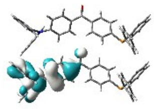
CDpP-G	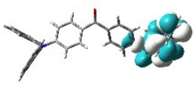	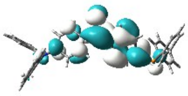	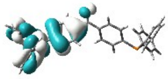	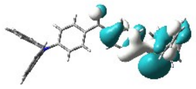
CDpP-G(dimer)	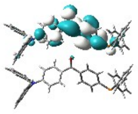	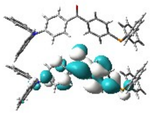	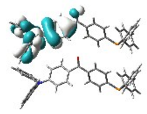	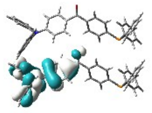

Table S3. Calculated energies of HOMO, LUMO, S_1 and T_1 orbitals and oscillator strength of transition for CDpP-B and CDpP-G both for monomer states and their dimer according to their single crystal structures.

Compounds		E_{S1} (eV)	E_{T1} (eV)	ΔE_{ST} (eV)	E_{HOMO} (eV)	E_{LUMO} (eV)	ΔE_g (eV)	$f_{(S_0\text{-}S_1)}$	$f_{(S_0\text{-}S_2)}$
CDpP-B	Monomer	3.26	2.58	0.68	-1.43	-5.09	3.66	0.45	0.03
	Dimer	2.89	2.57	0.32	-1.56	-1.88	3.32	<0.01	0.20
CDpP-G	Monomer	3.02	2.45	0.57	-1.66	-5.13	3.47	0.30	0.07
	Dimer	2.75	2.46	0.29	-1.77	-4.93	3.16	<0.01	0.18

* For dimer, the transition from S_0 to S_1 was corresponding to intermolecular charge transfer absorption,the other from S_0 to S_2 was corresponding to intramolecular charge transfer absorption

5 Single crystal data of CDpP(B) and CDpP(G)

The single-crystal X-ray data for CDpP-B and CDpP-G was obtained by an Oxford Diffraction Gemini S Ultra X-ray single-crystal diffractometer with graphite-monochromatized Cu-Kα radiation (λ = 1.54184 Å). The single-crystal structures were solved by Olex2 program and expanded with Fourier techniques. All non-H atoms of these compounds were refined with anisotropic thermal parameters. The hydrogen atoms were added in idealized positions and further refined with fixed geometry with respect to their carrier atoms. CCDC numbers for the single crystals of CDpP-B and CDpP-G are 1587616 and 1587617, respectively.

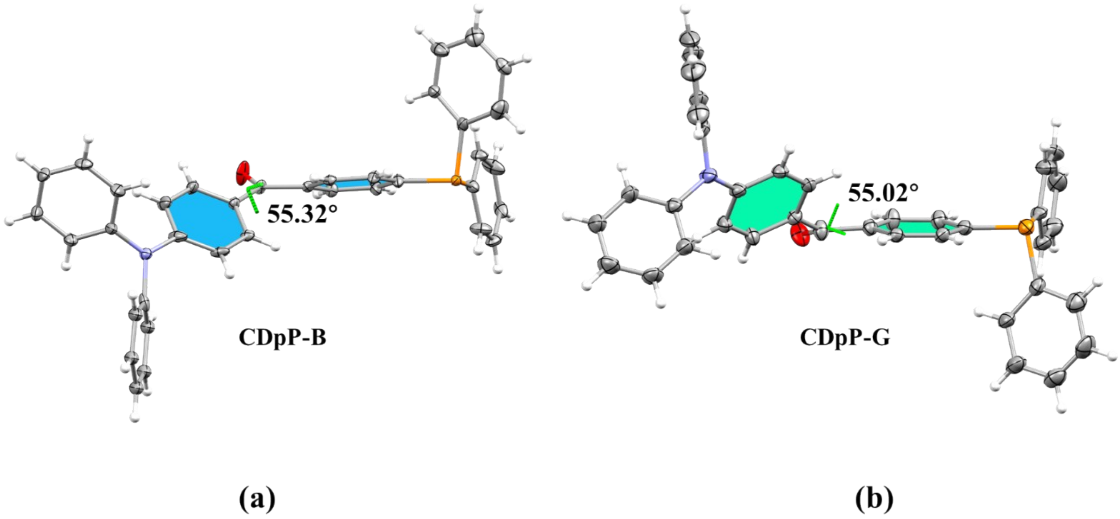

Figure S9 Dihedral angles between two phenylene groups across the carbonyl group of CDpP-B in two single crystals.

Crystal data for CDpP-B: $C_{37}H_{28}NOP$, Formula Weight = 533.57 g/mol, monoclinic, space group $P2_1/n$, T = 149.99(10) K, Z = 4, a = 21.3348(9) Å, b = 5.9392(2) Å, c = 23.3779(10) Å, α = 90 °, β = 102.485(4) °, γ = 90 °, V = 2892.2(2) Å^3, ρ_c = 1.225 g cm^{-3}, $\mu(Cu_{K\alpha})$ = 1.065 mm^{-1}, F(000) = 1120.0. Reflections collected 10043, Independent reflections 5719 (R_{int} = 0.0293). R_1 = 0.1496 (I ＞ 2σ(I)) and wR_2 = 0.3314, GOF = 1.062.

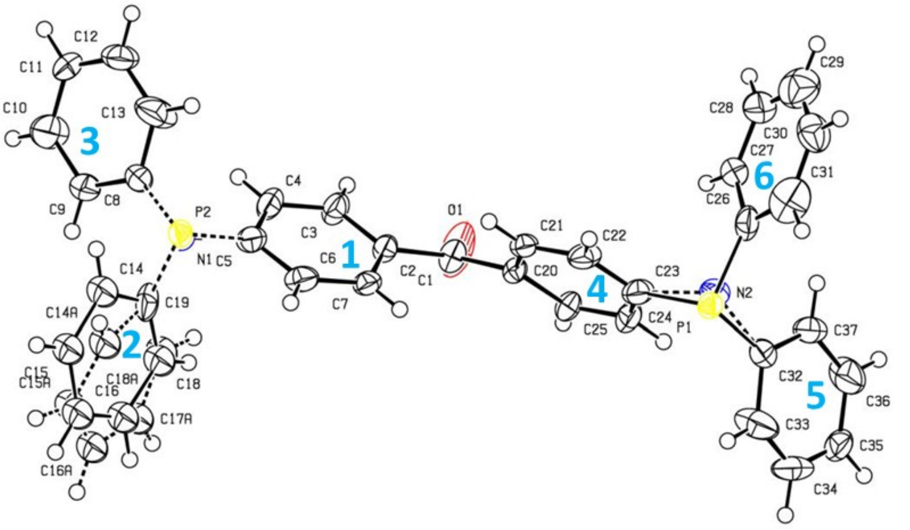

Figure S10 Single crystal strucutre for CDpP-B.

Table S4. Details of C-H···π interactions for CDpP-B.

C-H(I)···π(J)	$D_{H\cdots\pi}$(Å)	$D_{H\text{-}Perp}$(Å)	Gamma(°)	$A_{C\text{-}H\text{-}\pi}$ (°)	$D_{C\cdots\pi}$(Å)	$A_{C\text{-}H,\ \pi}$ (°)
C-H(10)···π(5)	2.76	2.74	6.80	153	3.61	60
C-H(12)···π(1)	2.78	2.76	8.16	157	3.66	74
C-H(15)···π(6)	2.93	2.75	19.76	146	3.73	75
C-H(28)···π(6)	2.95	2.91	9.54	133	3.65	53
C-H(34)···π(4)	2.82	2.77	10.19	149	3.64	69
C-H(36)···π(3)	2.96	2.90	10.91	147	3.65	63

* [π(J)] = Center of gravity of ring J (Plane number below); [H-Perp] = Perpendicular distance of H to ring plane J; [Gamma] = Angle between π-H vector and ring J normal; [C-H···π] = C-H-π angle (degrees); [C···π] = Distance of X to Cg (Angstrom); [C-H, π] = Angle of the C-H bond with the Pi-plane (i.e.' Perpendicular = 90 degrees, Parallel = 0 degrees)

Table S5. Bond distances (Å) for CDpP-B

**Atom**	**Atom**	**Length/Å**	**Atom**	**Atom**	**Length/Å**
P1	C26	1.771(6)	C37	C32	1.378(9)
P1	C32	1.822(7)	C37	C36	1.373(9)
P1	C23	1.839(6)	C32	C33	1.402(9)
O1	C1	1.236(7)	C32	N2	1.527(17)
C20	C21	1.392(7)	C23	N2	1.511(18)

C20	C1	1.486(7)	C11	C12	1.368(10)
C20	C25	1.400(7)	C11	C10	1.374(9)
C21	C22	1.377(8)	C8	N1	1.455(8)
C1	C2	1.479(7)	C8	C13	1.393(9)
C7	C2	1.386(8)	C8	P2	1.890(8)
C7	C6	1.379(8)	C12	C13	1.373(10)
C2	C3	1.402(7)	C18	C17	1.420(10)
C25	C24	1.381(8)	N1	C5	1.406(9)
C22	C23	1.389(9)	C30	C31	1.401(9)
C26	C27	1.381(7)	C30	C29	1.339(9)
C26	C31	1.443(9)	C34	C35	1.360(11)
C26	N2	1.738(19)	C34	C33	1.359(10)
C9	C8	1.360(9)	C5	C4	1.407(11)
C9	C10	1.377(9)	C5	P2	1.983(8)
C27	C28	1.366(7)	C35	C36	1.383(10)
C19	C18	1.357(14)	C16	C17	1.387(12)
C19	N1	1.525(9)	C16	C15	1.405(11)
C19	C14	1.369(10)	C28	C29	1.337(9)
C19	P2	1.604(8)	C14	C15	1.371(10)
C19	C18A	1.406(14)	C18A	C17A	1.404(14)
C19	C14A	1.439(14)	C17A	C16A	1.395(15)
C24	C23	1.419(10)	C16A	C15A	1.398(15)
C3	C4	1.372(8)	C15A	C14A	1.401(14)
C6	C5	1.411(10)			

Table S6. Bond angles for CDpP-B

Atom	Atom	Atom	Angle/°	Atom	Atom	Atom	Angle/°
C26	P1	C32	101.0(3)	C12	C11	C10	119.4(6)
C26	P1	C23	98.1(3)	C9	C8	N1	116.2(6)
C32	P1	C23	96.3(3)	C9	C8	C13	119.2(6)
C21	C20	C1	122.4(5)	C9	C8	P2	133.7(5)
C21	C20	C25	119.0(5)	C13	C8	N1	123.8(6)
C25	C20	C1	118.5(5)	C13	C8	P2	102.9(5)
C22	C21	C20	120.8(5)	C11	C12	C13	120.2(6)
O1	C1	C20	119.4(5)	C19	C18	C17	112.3(11)
O1	C1	C2	119.6(5)	C8	N1	C19	117.5(5)
C2	C1	C20	121.0(5)	C5	N1	C19	119.4(5)
C6	C7	C2	120.6(5)	C5	N1	C8	122.9(6)
C7	C2	C1	123.2(5)	C29	C30	C31	121.6(7)

C7	C2	C3	118.3(5)	C33	C34	C35	120.5(6)
C3	C2	C1	118.3(5)	C6	C5	P2	101.4(5)
C24	C25	C20	120.0(5)	N1	C5	C6	126.0(7)
C21	C22	C23	121.5(6)	N1	C5	C4	115.8(7)
C27	C26	P1	128.8(5)	C4	C5	C6	117.9(5)
C27	C26	C31	112.7(6)	C4	C5	P2	138.0(5)
C27	C26	N2	99.0(8)	C34	C35	C36	119.1(6)
C31	C26	P1	118.0(5)	C3	C4	C5	119.9(6)
C31	C26	N2	146.6(8)	C12	C13	C8	120.2(7)
C8	C9	C10	120.2(6)	C34	C33	C32	121.3(7)
C28	C27	C26	125.2(7)	C11	C10	C9	120.7(6)
C18	C19	N1	116.0(7)	C30	C31	C26	120.5(7)
C18	C19	C14	129.4(8)	C17	C16	C15	116.9(11)
C14	C19	N1	114.2(6)	C37	C36	C35	120.9(7)
C18A	C19	P2	137.9(12)	C29	C28	C27	120.4(8)
C18A	C19	C14A	99.2(16)	C28	C29	C30	119.5(8)
C14A	C19	P2	118.8(11)	C19	C14	C15	114.7(9)
C25	C24	C23	121.2(5)	C16	C17	C18	123.7(11)
C4	C3	C2	122.0(6)	C14	C15	C16	122.8(11)
C7	C6	C5	121.3(6)	C19	P2	C8	93.2(4)
C36	C37	C32	120.4(6)	C19	P2	C5	89.1(4)
C37	C32	P1	129.9(5)	C8	P2	C5	80.9(4)
C37	C32	C33	117.7(6)	C32	N2	C26	116.1(11)
C37	C32	N2	103.7(8)	C23	N2	C26	113.9(10)
C33	C32	P1	112.2(5)	C23	N2	C32	127.6(12)
C33	C32	N2	134.4(8)	C17A	C18A	C19	133(2)
C22	C23	P1	113.4(5)	C16A	C17A	C18A	119(2)
C22	C23	C24	117.5(5)	C17A	C16A	C15A	117(2)
C22	C23	N2	135.9(9)	C16A	C15A	C14A	115(3)
C24	C23	P1	128.9(5)	C15A	C14A	C19	135(2)
C24	C23	N2	102.5(9)				

Crystal data for CDpP-G: $C_{37}H_{28}NOP$, Formula Weight = 533.57 g/mol, monoclinic, space group Cc, T = 272(4) K, Z = 4, a = 23.584(4) Å, b = 6.0025(7) Å, c = 21.526(3) Å, α = 90 °, β = 102.573(14) °, γ = 90 °, V = 2974.2(7) Å^3, ρ_c = 1.192 g cm^{-3}, $\mu(Cu_{K\alpha})$ = 1.036 mm^{-1}, F(000) = 1120.0. Reflections collected 4795, Independent reflections 3619 (R_{int} = 0.0246), R_1 = 0.0872 (I ＞ 2σ(I)) and wR_2 = 0.2447, GOF = 1.372.

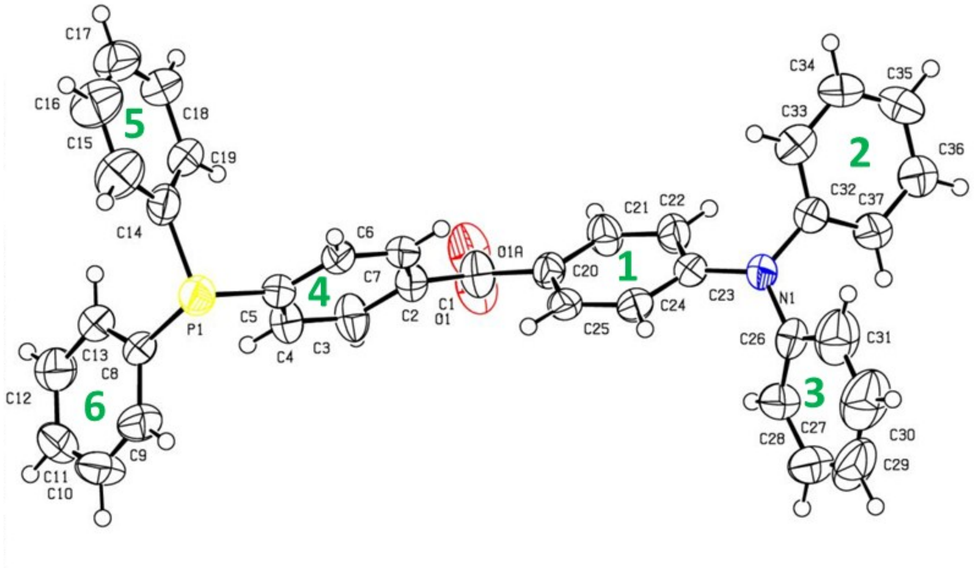

Figure S11 Single crystal strucutre for CDpP-G.

Table S7. Details of C-H···π interactions for CDpP-G.

C-H(I)···π(J)	$D_{H\cdots\pi}$(Å)	$D_{H\text{-}Perp}$(Å)	Gamma(°)	$A_{C\text{-}H\text{-}\pi}$ (°)	$D_{C\cdots\pi}$(Å)	$A_{C\text{-}H,\ \pi}$ (°)
C-H(10)···π(1)	2.98	2.88	14.78	147	3.79	70
C-H(17)···π(1)	2.96	2.80	19.26	135	3.68	56
C-H(34)···π(4)	2.69	2.68	4.43	161	3.58	74
C-H(36)···π(6)	2.83	2.81	6.02	154	3.69	59

* [π(J)] = Center of gravity of ring J (Plane number below); [H-Perp] = Perpendicular distance of H to ring plane J; [Gamma] = Angle between π-H vector and ring J normal; [C-H···π] = C-H-π angle (degrees); [C···π] = Distance of X to Cg (Angstrom); [C-H, π] = Angle of the C-H bond with the Pi-plane (i.e.' Perpendicular = 90 degrees, Parallel = 0 degrees)

Table S8. Bond distances (Å) for CDpP-G

**Atom**	**Atom**	**Length/Å**	**Atom**	**Atom**	**Length/Å**
P1	C8	1.806(7)	C26	C31	1.396(13)
P1	C5	1.837(7)	C5	C6	1.403(9)
P1	C14	1.816(8)	N1	C32	1.457(9)
C20	C25	1.382(9)	C34	C35	1.361(16)
C20	C1	1.484(9)	C34	C33	1.379(13)

C20	C21	1.408(9)	C27	C28	1.417(15)
O1	C1	1.260(14)	C21	C22	1.360(10)
C25	C24	1.390(10)	C14	C19	1.394(12)
C2	C7	1.416(9)	C14	C15	1.385(13)
C2	C1	1.472(10)	C32	C33	1.372(11)
C2	C3	1.408(9)	C32	C37	1.368(12)
C7	C6	1.376(10)	C35	C36	1.380(13)
C24	C23	1.399(10)	C36	C37	1.379(12)
C4	C5	1.415(10)	C19	C18	1.375(11)
C4	C3	1.393(10)	C18	C17	1.346(15)
C23	N1	1.404(9)	C12	C11	1.382(14)
C23	C22	1.412(10)	C31	C30	1.324(18)
C8	C13	1.404(12)	C29	C30	1.36(3)
C8	C9	1.400(9)	C29	C28	1.41(3)
C1	O1A	1.257(16)	C17	C16	1.344(17)
C13	C12	1.387(12)	C16	C15	1.374(15)
C26	N1	1.424(9)	C9	C10	1.389(16)
C26	C27	1.380(11)	C11	C10	1.369(19)

Table S9. Bond angles for CDpP-G

Atom	Atom	Atom	Angle/°	Atom	Atom	Atom	Angle/°
C8	P1	C5	99.9(3)	C23	N1	C26	119.7(6)
C8	P1	C14	103.6(3)	C23	N1	C32	119.3(6)
C14	P1	C5	100.2(3)	C26	N1	C32	118.1(6)
C25	C20	C1	123.8(6)	C35	C34	C33	120.4(7)
C25	C20	C21	118.3(6)	C26	C27	C28	117.5(11)
C21	C20	C1	117.8(6)	C22	C21	C20	122.0(6)
C20	C25	C24	120.4(6)	C19	C14	P1	124.8(6)
C7	C2	C1	123.3(6)	C15	C14	P1	120.4(7)
C3	C2	C7	117.6(6)	C15	C14	C19	114.3(8)
C3	C2	C1	119.0(6)	C4	C3	C2	120.6(6)
C6	C7	C2	121.8(6)	C33	C32	N1	120.2(8)
C25	C24	C23	121.0(6)	C37	C32	N1	120.0(6)
C3	C4	C5	121.3(6)	C37	C32	C33	119.7(7)
C24	C23	N1	121.4(7)	C21	C22	C23	119.9(6)
C24	C23	C22	118.3(6)	C34	C35	C36	119.3(8)
N1	C23	C22	120.3(7)	C37	C36	C35	120.4(9)
C13	C8	P1	127.0(5)	C32	C33	C34	120.3(9)
C9	C8	P1	115.4(6)	C18	C19	C14	122.1(9)

C9	C8	C13	117.5(8)	C17	C18	C19	120.6(11)
O1	C1	C20	117.7(13)	C11	C12	C13	119.5(10)
O1	C1	C2	120.0(13)	C30	C31	C26	119.7(13)
C2	C1	C20	120.6(6)	C30	C29	C28	118.1(10)
O1A	C1	C20	120.9(17)	C16	C17	C18	119.7(9)
O1A	C1	C2	115.8(17)	C31	C30	C29	123.3(13)
C12	C13	C8	121.7(7)	C17	C16	C15	120.0(9)
C27	C26	N1	120.2(7)	C10	C9	C8	120.0(9)
C27	C26	C31	120.8(9)	C10	C11	C12	119.7(9)
C31	C26	N1	118.7(8)	C16	C15	C14	123.1(10)
C4	C5	P1	125.9(5)	C11	C10	C9	121.5(8)
C6	C5	P1	116.3(5)	C29	C28	C27	120.2(13)
C6	C5	C4	117.8(6)	C32	C37	C36	119.9(7)
C7	C6	C5	121.0(6)				

6 Photophysical data to conform the energy levels in the photodynamic scheme

For CDpP-B, the energy levels for the LE state, LE’ state were conformed by the global analysis of femtosecond transient emission spectra. Similarly, the LE state and ^{1}TICT states for CDpP-G could also be calculated according to the global analysis of femtosecond transient emission spectra. The energy level for ^{3}TICT was calculated according to the emission spectra for CDpP-G at 127 K as shown in the following spectra. At 127 K, the excited states could be decay form the ^{3}TICT and the emission from ^{3}LE were not shown. The ^{3}LE emission bands for both CDpP-B and CDpP-G could be detected at 77 K.

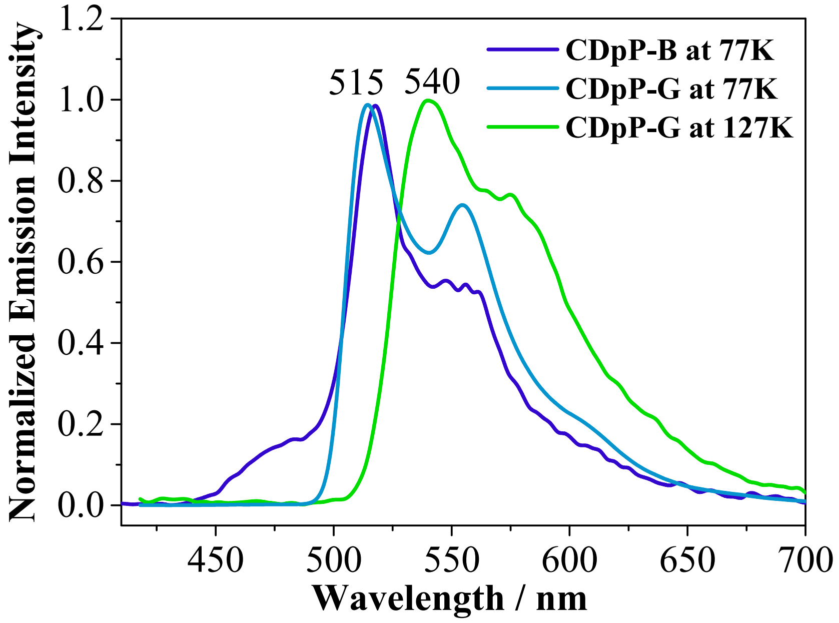

Figure S12 Emission spectra for CDpP-B at 77 K, CDpP-G at 77 K and 127 K.

Reference:

[1] (a) S. Xu, T. Liu, Y. Mu, Y. F. Wang, Z. Chi, C. C. Lo, S. Liu, Y. Zhang, A. Lien, J. Xu, *Angew. Chem., Int. Ed.* 2015, **54**, 874-878. (b) Z. Xie, C. Chen, S. Xu, J. Li, Y. Zhang, S. Liu, J. Xu, Z. Chi, *Angew. Chem., Int. Ed.* 2015, **54**, 7181-7184.
